# Unusual presentation of *Ascaris lumbricoides* in the urinary tract: a case report

**DOI:** 10.1093/bjrcr/uaaf001

**Published:** 2025-01-16

**Authors:** Zaid A Sowaity, Ammar A Zamareh, Tayseer N Sabooh, Amjed T Sowaity, Yazan F Khdour, Mutaz A A Atmeza

**Affiliations:** Faculty of Medicine, Al Quds University, Jerusalem, Palestine; Faculty of Medicine, Al Quds University, Jerusalem, Palestine; Faculty of Medicine, Al Quds University, Jerusalem, Palestine; Department of Urology, Alia Hospital, Hebron, Palestine; Faculty of Medicine, Alexandria University, Alexandria, Egypt; Department of Radiology, Dura Hospital, Hebron, Palestine

**Keywords:** *Ascaris lumbricoides*, urinary worm, ureteroscopy, urologic diseases/parasitology, roundworms, ureteral parasite

## Abstract

*Ascaris lumbricoides* is one of the most well-known helminthic parasites affecting humans. Ascariasis is prevalent in developing countries where inadequate water, sanitation, and hygiene facilitate human-to-human transmission. In this report, we present a case of a 20-year-old male who arrived at the emergency room with severe right flank pain, high-grade fever, and recurrent vomiting. Diagnostic evaluations were conducted, including a complete blood count test, urinalysis, stool analysis, abdominal ultrasound, and CT scan. The final diagnosis was *A lumbricoides* found in the distal part of the ureter. A ureteroscopy procedure confirmed the diagnosis and extracted the worm, which measured 6 cm in length, had a brown colour, and exhibited a tight elastic consistency. While Ascaris is commonly found in the gastrointestinal tract, its occurrence in the urinary tract is an extremely rare phenomenon. In our case, the most likely explanation is that the Ascaris accessed the distal ureter through retrograde migration, wherein the worm traverses from the bladder into the ureter.

## Introduction

Ascariasis is a parasite infection that is widely spread throughout the world, particularly in tropical and subtropical regions.^1^ Globally, about 1.5 billion people are affected by *Ascaris lumbricoides* infection.^2^ Ascariasis is prevalent in developing countries where a lack of adequate water, sanitation, and hygiene facilitates human-to-human transmission. Roundworm infection is transmitted through faeces that contaminate the environment with parasite eggs. Adult females lay about 200 000 eggs per day, which are then released into the soil in their feces.^3^ Ascaris is infected by ingesting embryonated eggs from raw vegetables, water, or soil-contaminated hands. Fertilized eggs hatch in the gut, which makes the gastrointestinal tract the most common site of Ascaris. Released larvae penetrate the intestinal wall into the right heart, the pulmonary circulation, and then into the alveoli. After the larvae are coughed up by the host, they are swallowed back into the gut, where they develop into adults. When adult worms migrate, they can cause severe clinical issues such as acute pancreatitis, acute cholecystitis, liver abscess, intestinal obstruction, and even perforation.^1^ The most common location of Ascaris is the gastrointestinal tract; Ascaris in the urinary tract is an extremely rare phenomenon.^4^ This presentation is for the parasite that may be challenging to diagnose. Imaging plays a role in detecting and confirming these atypical cases.

## Case presentation

**Figure 1.**
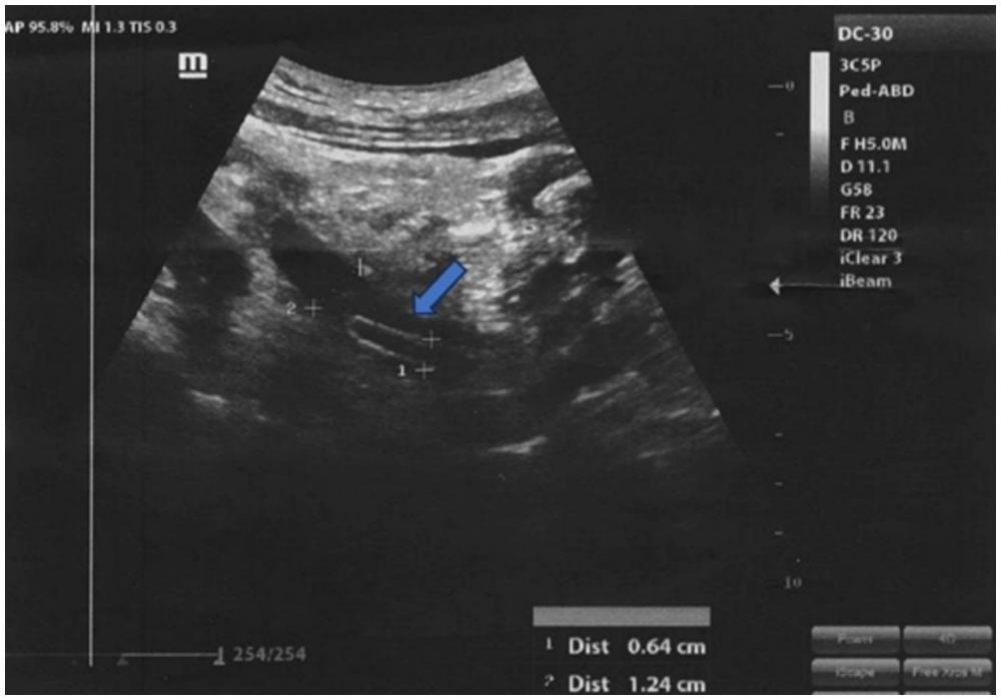
Renal ultrasound of the right kidney showed a cylindrical structure measuring 6 mm in diameter and 6 cm in length in the distal part of the right ureter.

A 20-year-old male working as a farmer, free of past medical and surgical history was admitted to the emergency department complaining of severe colic pain in nature in the right flank region, radiating to the surrounding area and groin. The pain is associated with high-grade fever and recurrent vomiting. He reported painless hematuria, fever, and flu-like symptoms 2 weeks before admission to the hospital. Fever and flu-like symptoms had been treated with the use of analgesia. He had no previous history of urinary stone disease or urinary tract infections. Physical examination showed tenderness in the right groin and positive right costovertebral angle tenderness, otherwise, it was normal. A complete blood count test, urinalysis, stool analysis, and abdominal ultrasound were performed. Laboratory analysis showed moderate leucocytosis of 20 000/mm^3^ (normal range: 4.5-11 × 10^3^/mm^3^), relative neutrophilia, and relative lymphopenia. The serum creatinine was normal. Urinalysis showed +3 hematuria and calcium oxalate crystals. The stool analysis showed Entamoeba histolytica present, with no ova or worms detected, and the rest of the stool analysis was normal. Abdominal ultrasound showed a hyperechoic cylindrical structure measuring 6 mm in diameter and 6 cm in length in the distal part of the right ureter involving the ureterovesical junction (UVJ) associated with minimal right-sided hydronephrosis and a mild increase in the echogenicity of the right kidney (Figure 1). There is no definite renal stone; otherwise, both kidneys are normal in size, site, and shape. Doppler imaging was not performed.

**Figure 2.**
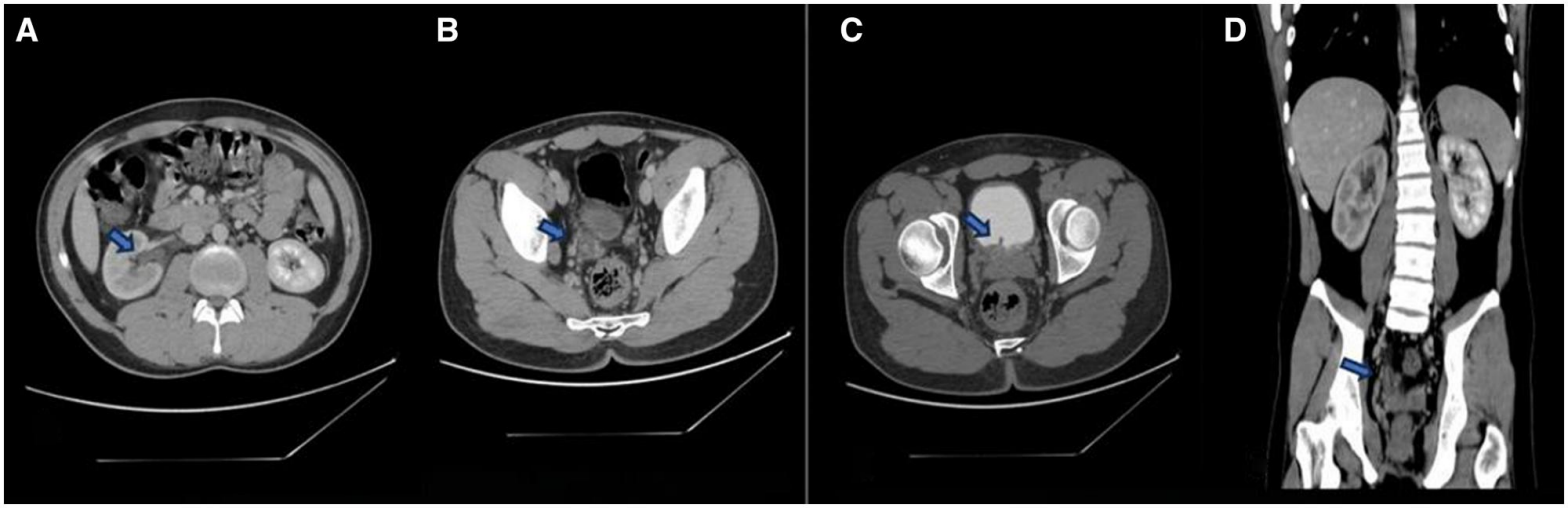
(A) Axial image at a level right renal pelvis on venous phase showing mild hydronephrosis abdominal perinephric fat stranding due to the distal ureteric obstruction it also shows hypo enhancement of the right kidney compared to the left side. (B) Axial image at the level of the distal part of the right ureter showing dilated ureter and rounded filling defect representing the obstructing parasite. (C) Axial CT image at the level of VUJ at 10 min delay phase showing the parasite partially passing through the vesicoureteral junction. (D) Coronal CT image showing the cylindrical structure at the distal ureter.

**Figure 3.**
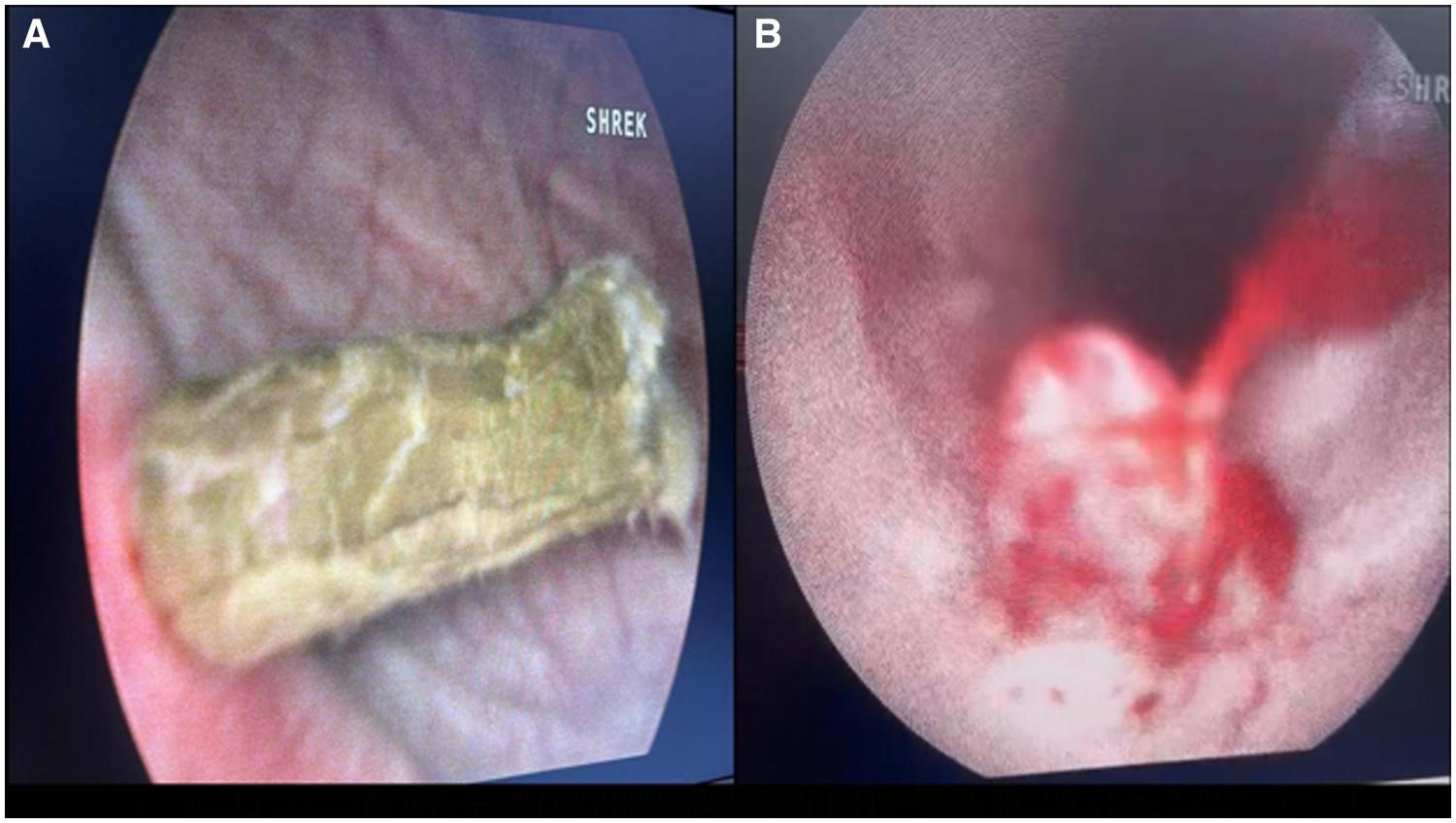
The ureteroscope revealed a wax-like structure (a worm) at the ureterovesical junction during extraction.

**Figure 4.**
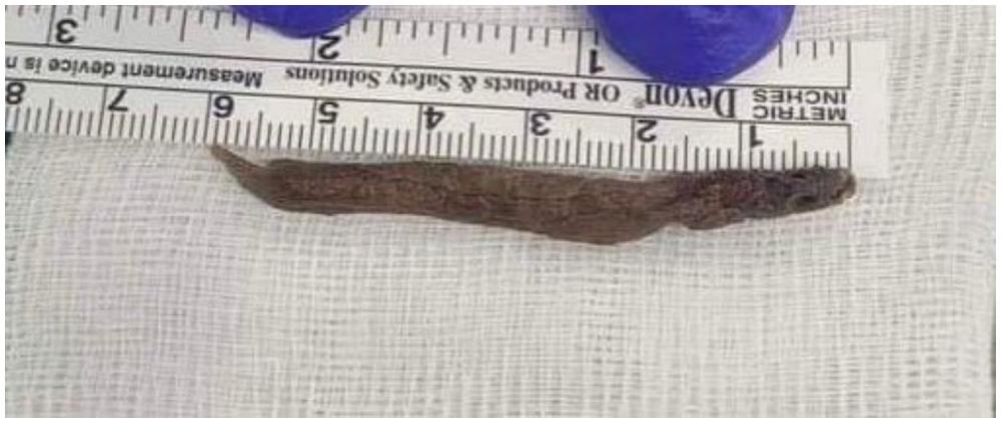
The Ascaris lumbricoides after ureteroscopic extraction with 6 cm length.

He was hospitalized in the urology department; conservative treatment was initially pursued. A contrast CT scan was done, which showed a 6-cm tubular-shaped structure (a foreign body) located at the distal ureter about 2.5 cm inside the bladder (Figure 2). The structure shows soft tissue density in the wall and fluid density in the inner aspect, causing minimal right-sided hydroureteronephrosis (Figure 3). An axial image at the level of the distal part of the right ureter shows a dilated ureter and rounded filling defect representing the obstructing parasite (Figure 4). Axial CT image at the level of UVJ at 10 min delay phase showing the parasite partially passing through the vesicoureteral junction (Figure 2). Otherwise, the right kidney is normal in size, echotexture, and cortical thickness with no stones or masses. There is no evidence of a fistula between the gastrointestinal and urinary tract.

A ureteroscopy was performed at the hospital. During the procedure, a 6-mm wax-like structure was discovered in the distal third of the right ureter, accompanied by a significant amount of pus. Additionally, a polyp-like projection was observed within the ureteroscope view (Figure 3). The extracted object measured 6 cm in length, had a brown colouration, was not moving structure, and displayed a tight elastic consistency (Figure 4). To facilitate pus drainage, a Double J (ureteral stenting) was inserted. Subsequently, the dead specimen underwent pathological review, identifying it as *A lumbricoides*. The patient received a 400 mg albendazole tablet once daily for 3 days and experienced an uneventful recovery.

## Discussion

This case report discusses an unusual instance of *A lumbricoides* found in the distal ureter of the urinary tract. Since this parasite usually inhabits the gastrointestinal tract, detecting and removing the worm from this site raises important questions regarding its pathophysiology.^5^ The maturation of the Ascaris larva into the adult worm form is only possible in the gastrointestinal system.

There are only 2 possible explanations on how this worm can be introduced into the urinary system, especially in the ureter involving either the creation of a fistula connecting the ureter with the gastrointestinal tract or the retrograde ascending of this parasite through the urinary system.

According to the first theory, the cause of fistula between neighbouring organs may arise from infections, chronic inflammation, or surgical procedures. However, another potential reason for its occurrence in the ureter could be retrograde ascending, in which the worm travels upward from the bladder or the urethra against the urinary flow. Moreover, the ureteroscopy has ruled out the presence of a fistula, which further emphasizes the unusual nature of the worm’s route to the ureter.

The 2 possible reasons demonstrate the complexity and singularity of this case for the worm’s existence. How this also emphasizes the need for a deeper understanding of parasites like Ascaris find their way into atypical areas of the human body, challenging conventional diagnostic and therapeutic approaches.

Gaining insight into these explanations could enhance diagnostic and treatment strategies for similar patients. Moreover, this case underscores the importance of considering parasitic infections during differential diagnoses, particularly in regions with *A lumbricoides*.

Definitive diagnosis of *A lumbricoides* involves identifying adult worms expelled from any body orifice or detecting their eggs in stool, urine, vomitus, sputum, or small bowel aspirate under a microscope. Imaging plays a crucial role in identifying this parasite in unusual positions. Ultrasound typically displays the worms as long echogenic structures with a central longitudinal echogenic tube representing the alimentary canal. On CT scans, worms are seen as elongated or rounded filling defects in the contrast-filled lumen, and multidetector CT can reveal entire worm lengths or clumps in heavily infested individuals. When correlated with clinical presentation, these imaging techniques are fundamental in confirming the presence of ascarids in atypical locations such as the urinary tract system.^6^

The effectiveness of the anthelmintic drug in treating parasitic infections is evident, even when they manifest in unconventional anatomical sites, as shown by the patient’s successful treatment with albendazole and their smooth recovery. This case provides valuable insights that encourage careful evaluation and vigilance in managing similar cases while making differential diagnoses, especially in endemic regions for *A lumbricoides*. The patient’s positive outcome following albendazole treatment further demonstrates the efficacy of this anthelmintic medication against parasites in unusual locations.

## Conclusion

The presence of *A lumbricoides* in the ureter is highly unusual and typically associated with regions lacking sufficient water, sanitation, and hygiene. Enhancing environmental standards is essential to curbing the spread of this parasite. Patients experiencing symptoms such as flank pain, fever, vomiting, and hematuria in low-hygiene settings should remain vigilant, considering a range of potential causes beyond the usual suspects like renal stones or infection. Timely identification and treatment of Ascaris can help prevent undesirable complications.

## Learning points

- *Ascaris lumbricoides* can present in atypical locations, such as the urinary tract.
- Imaging techniques such as ultrasound and CT scans play a critical role in diagnosing unusual parasitic infections by identifying the worm’s distinct structure in the urinary system.
- Parasitic infections should be considered in the differential diagnosis of flank pain and hematuria, particularly in endemic regions or low-hygiene settings.

## Data Availability

The corresponding author will provide the data sets used and/or analysed during the current study upon reasonable request.
